# Exome Sequencing Identified a Novel *FBN2* Mutation in a Chinese Family with Congenital Contractural Arachnodactyly

**DOI:** 10.3390/ijms18040626

**Published:** 2017-04-05

**Authors:** Guoling You, Bailing Zu, Bo Wang, Zhigang Wang, Yunlan Xu, Qihua Fu

**Affiliations:** 1 Department of Laboratory Medicine, Shanghai Children’s Medical Center, Shanghai Jiao Tong University School of Medicine, Shanghai 200127, China; youguoling@126.com (G.Y.); wangbao@scmc.com.cn (B.W.); 2 Department of Laboratory Medicine, Shanghai East Hospital, Tongji University School of Medicine, Shanghai 200127, China; zubailing@126.com; 3 Department of Clinical Laboratory, Shanghai Pudong Hospital, Fudan University Pudong Medical Center, Shanghai 200127, China; 4 Department of Pediatric Orthopedics, Shanghai Children’s Medical Center, Shanghai Jiao Tong University School of Medicine, Shanghai 200127, China; wangzhigang@scmc.com.cn

**Keywords:** congenital contractural arachnodactyly, exome sequencing, fibrillin-2 (*FBN2*) gene

## Abstract

Congenital contractural arachnodactyly (CCA) is an autosomal dominant disorder of connective tissue. CCA is characterized by arachnodactyly, camptodactyly, contrature of major joints, scoliosis, pectus deformities, and crumpled ears. The present study aimed to identify the genetic cause of a three-generation Chinese family with CCA. We successfully identified a novel missense mutation p.G1145D in the fibrillin-2 (*FBN2*) gene as the pathogenic mutation by whole exome sequencing (WES). The p.G1145D mutation occurs in the 12th calcium-binding epidermal growth factor-like (cbEGF) domain. The p.G1145D mutation caused a hydrophobic to hydrophilic substitution, altering the amino acid property from neutral to acidic. Three-dimensional structural analysis showed that this mutation could alter the conformation of the residue side chain, thereby producing steric clashes with spatially adjacent residues, disrupting the formation of H bonds and causing folding destabilization. Therefore, this amino acid appears to play an important role in the structure and function of *FBN2*. Our results may also provide new insights into the cause and diagnosis of CCA and may have implications for genetic counseling and clinical management.

## 1. Introduction

Congenital contractural arachnodactyly (CCA, Online Mendelian Inheritance in Man (OMIM), 121050), also known as Beals–Hecht syndrome, is an autosomal dominant connective tissue disorder [1], with extremely rare incidence [2]. The incidence of CCA is unknown, but appears to be lower than that of the Marfan syndrome (MFS). The prevalence of CCA is difficult to estimate considering the overlap in phenotype with MFS. CCA can be divided into classical CCA and severe/lethal CCA [3]. Classical CCA is characterized by arachnodactyly, camptodactyly, contracture of major joints (hips, knees, ankles, or elbows), scoliosis, pectus deformities, and crumpled ears [4]. Severe/lethal CCA is a rare form of CCA that is characterized by multiple cardiovascular (aortic root dilatation) and gastrointestinal anomalies in addition to the typical skeletal features [5]. CCA, which appears to be fully penetrant, exhibits no specific geographic or ethnic predilection; however, germline mosaicism has been observed [6].

Although CCA and MFS are often difficult to differentiate clinically because of their phenotypic similarities, CCA can be distinguished from MFS genetically: the fibrillin-1 (*FBN1*) gene defects on 15q15-21.3 may cause MFS, while the fibrillin-2 (*FBN2*) gene on 5q23-31 is linked to CCA [1,7]. *FBN1* and *FBN2* are highly homologous genes with 65 exons that encode the large, cysteine-rich extracellular matrix (ECM) glycoproteins fibrillin-1 and fibrillin-2, respectively. Both fibrillins form microfibrils, and they modulate the concentration, presentation, and activation (bioavailability) of transforming growth factor β (TGFβ) and bone morphogenic protein (BMP) complexes locally [8,9]. Gerhard et al. demonstrated the important role of fibrillin-2 for early postnatal muscle development; they found that Fbn2 null mice had severe defects in their musculature [10]. In other words, a missense mutation in the *FBN2* gene, which leads to the folding destabilization and reduced structural integrity of fibrillin-2, may cause the muscle defects underlying contractural arachnodactyly. Moreover, fibrillin-2 assemblies promote apoptosis of naive mesenchymal cells in the interdigital matrix by locally concentrating BMP complexes [8,11]. A missense mutation in *FBN2* might perturb interdigital matrix organization and prevent mesenchymal cell death, resulting in the tissue-level outcome of fused digits.

The *FBN2* gene is >28 kb, and, for such a large gene, a relatively small number of mutations have been identified in it to date. Approximately 85 mutations in the *FBN2* gene have been identified as pathogenic, as listed in the Human Gene Mutation Database (HGMD) Professional database. These mutations vary from point mutations to gross deletions. A total of 68% of these pathogenic mutations are missense mutations, while 17.6% are splicing mutations. More than 1000 reported *FBN1* mutations are spread throughout the gene [12], while the majority of *FBN2* mutations associated with CCA occur in Exons 24–35, which encode the calcium-binding epidermal growth factor-like (cbEGF) domains. The homologous region in *FBN1* (Exons 23–35) contains clusters of mutations, implicated in severe forms of MFS, including neonatal MFS (severe early-onset disease). All of these mutations reduce the amount of fibrillin-2 available to form microfibrils. Decreased microfibril formation reduces the elasticity of fibers, which leads to the symptoms of CCA.

In the present study, we described a novel mutation seen in a family with three generations of CCA. We performed whole exome sequencing (WES) in two affected individuals to screen the pathogenic gene for this pedigree and identified a novel mutation in the *FBN2* gene in all affected family members.

## 2. Results

### 2.1. The Clinical Phenotypes of the Congenital Contractural Arachnodactyly (CCA) Pedigree

**Figure 1:**
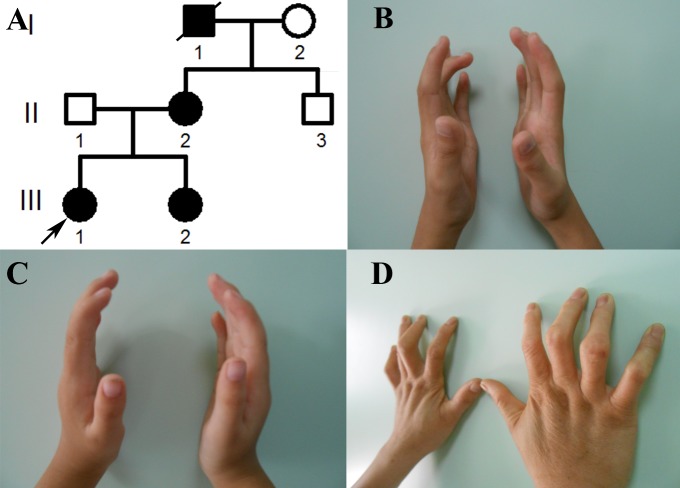
Pedigree information and clinical features of patients with congenital contractural arachnodactyly (CCA). (**A**) Pedigree of the family with CCA. Generations are shown as I to III. Males are indicated by squares, females by circles, affected members by a shaded black square or circles, and the proband (III-1) by an arrow. Shaded black square with slash indicates death; (**B**) Photographs of findings in the proband; (**C**) Photographs of findings in the proband’s sister; (**D**) Photographs of findings in the proband’s mother.

A three-generation pedigree with autosomal dominant CCA was enrolled in this study (Figure 1A). The clinical features of the three patients in this family who participated in this study are shown in Table 1. The proband (III-1), a fifteen-year-old girl, presented with slender, contractural fingers and toes (Figure 1B). Her parents are not consanguineous. Her mother (II-2) (Figure 1D) and her younger sister (III-2) (Figure 1C) also presented with slender, contractural fingers. Her grandfather died at the age of 67. In addition to arachnodactyly and camptodactyly, he also exhibited large-joint contracture. No other pathological features, such as neurological abnormalities, cardiovascular abnormalities, external ear malformations, or eye abnormalities were observed in the proband or other affected family members. Intrafamilial variation in phenotypic expression was modest.

### 2.2. Mutation Screening for the CCA Pedigree

A total of 83,560,000 and 81,250,000 reads were obtained from the sequencing results of two samples, respectively. The average target coverages were 57.8× and 52.6×, respectively. Of these, an average of 97% of the reads had Phred-like quality scores (Q scores) of greater than 20% and 90% of the reads had Q scores of greater than 30, which is a sufficient depth to interrogate the exons for mutations. There were 34,330 and 32,189 single nucleotide variations (SNVs) in the exome prior to filtering, with 6194 and 5014 SNVs occurring in coding regions, respectively.

**Figure 2:**
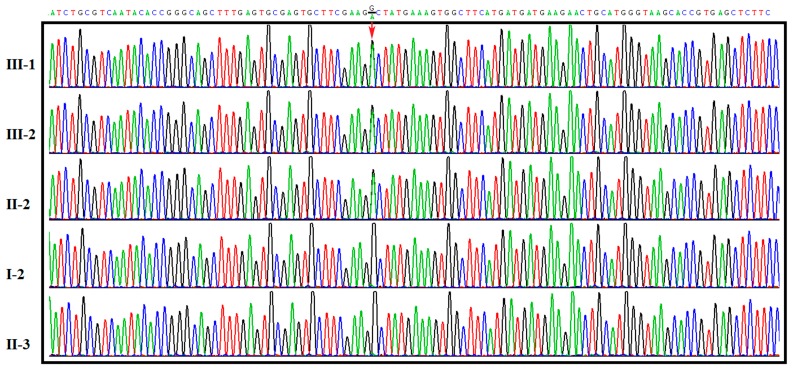
Sanger sequencing to confirm the fibrillin-2 (*FBN2*) c.3434G>A mutation in the CCA family members. The mutation is marked by a red arrow.

Variants with a minor allele frequency (MAF) ≥1% in the data from the 1000 Genomes Project were excluded, since the CCA-causing variant should be rare in the general population. As we expected the disease-causing variant to be shared by all sequenced and affected individuals, all variants not fulfilling this criterion were excluded. Given the apparent autosomal dominant mode of inheritance in this family, all homozygous sequence variants were further excluded from the analysis of CCA-causing mutations. In addition, we removed all synonymous variants. The variants were analyzed in silico with the Scale Invariant. Feature Transform (SIFT), Polymorphism Phenotyping v2 (PolyPhen-2), MutationTaster, Rare Exome Variant Ensemble Learner (REVEL) and Mendelian Clinically Applicable Pathogenicity (M-CAP) to predict of deleterious nonsynonymous SNVs for human diseases. Finally, a solitary heterozygous missense variant, c.3434G>A (NM_001999) in Exon 26 of the *FBN2* gene (Figure 2), was identified in the proband (III-1) based on the OMIM, a Gene Ontology (GO) analysis, Kyoto Encyclopedia of Genes and Genomes (KEGG) pathway analysis, and the HGMD. This mutation converted amino acid 1145 from glycine to aspartic acid (p.G1145D). The mutation was confirmed by Sanger sequencing. This missense mutation was absent from the HGMD, Single Nucleotide Polymorphism (SNP) database (dbSNP), 1000 Genomes Project (TGP) database and ESP6500 database, Exome Aggregation Consortium (ExAC) database, ClinVar database and whole genome sequencing data of 81 unrelated person. Additionally, to our knowledge, this mutation has not been described in the Universal Mutation Database FBN2 (UMD-FBN2) database or reported in any published literature.

### 2.3. Co-Segregation Analysis and Mutation Validation

The p.G1145D mutation was also identified in other affected family members (II-2 and III-2) by direct sequencing (Figure 2). The missense mutation was not found in unaffected family members (I-2 and II-3) (Figure 2). The p.G1145D mutation in the *FBN2* gene showed complete co-segregation in the family. In addition, we further confirmed the absence of this mutation in the *FBN2* gene in 200 unrelated, ethnically and geographically matched controls, excluding the possibility of SNP.

### 2.4. In Silico Functional Analysis

**Figure 3:**
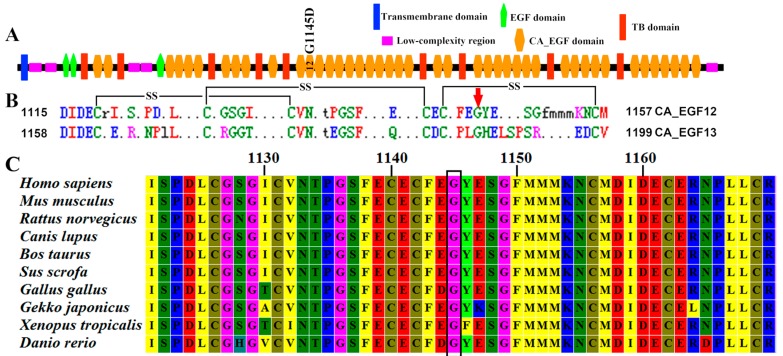
Domain structure and sequence conservation analysis of the *FBN2* protein. (**A**) The putative structural domains and variants identified are located in CA_epidermal Growth Factor-Like (CA_EGF) Domain 12; (**B**) Multiple sequence alignment of CA_EGF Motifs 12–13. Disulfide bonds are shown by black lines (SS). Locations for Gly1145 are shown by red arrow; (**C**) Phylogenetic comparison of *FBN2* protein across species. Position of the mutation within a highly conserved region indicated with black box. TB: transforming growth factor β binding protein-like.

To better understand the effect of the mutation on protein function, we analyzed the mutation in silico with SIFT, PolyPhen-2, MutationTaster, REVEL, and M-CAP to predict deleterious SNVs for human diseases. Based on this analysis, the G1145D substitution was predicted to be deleterious based on SIFT score (<0.05), rated as “probably damaging” by PolyPhen2 with a score of 1.0, predicted as “disease causing” by MutationTaster analysis with a score of 1.0, classified as “pathogenic” by REVEL analysis with a score of 0.943 (close to 1.0) and by M-CAP analysis with a score of 0.424 (>0.025). An alignment of *FBN2* protein sequences revealed that this position is highly conserved among many different species (Figure 3C). Thus, this amino acid appears to play an important role in the structure and function of fibrillin-2. 

**Figure 4:**
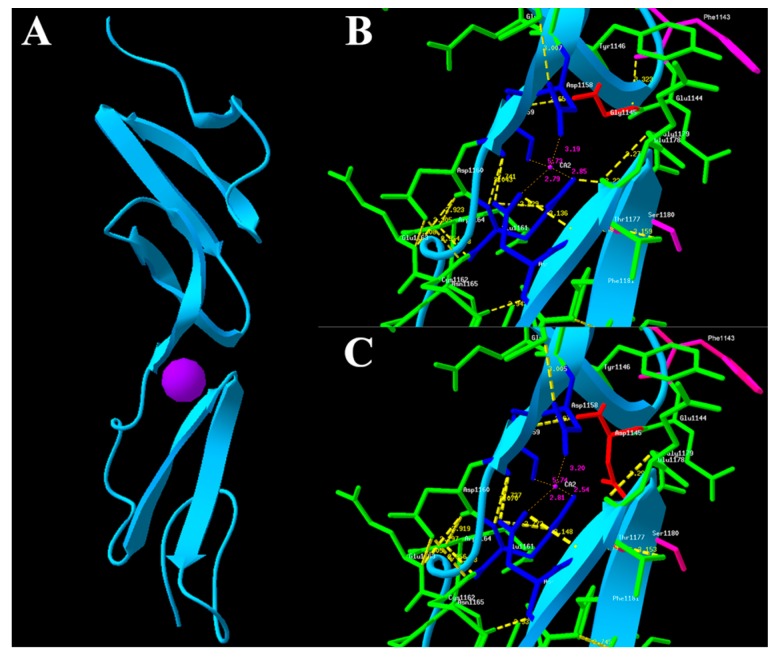
Homology modeling of wild-type and mutant *FBN2* variants. (**A**) Modeled structure of cbEGF Domains 12 and 13 of the *FBN2* protein; (**B**) Neighboring residues of Gly1145 in the wild type of *FBN2*. Gly1145 is shown in red; (**C**) Neighboring residues of Asp1145 in mutant *FBN2*. Asp1145 is shown in red. Predicted H bonds are indicated by yellow dashed lines. Ca^2+^ is depicted as purple ball.

The fibrillin-2 is comprised of 2912 residues and contains 3 epidermal growth factor-like (EGF) domains, 9 transforming growth factor β binding protein-like (TB) domains, and 43 cbEGF domains (Figure 3A). The native protein fold of each cbEGF-like domain is maintained by six conserved cysteine residues, which form three disulfide bridges to support protein stability (Figure 3B). The Ca^2+^ chelation occurs in cbEGF domain pairs (Figure 4A), and this pair forms a four-sheet bundle to make a hydrophobic core. The G1145D mutation caused converted amino acid from glycine to aspartic acid. However, the conserved glycine residue of the short antiparallel β-sheet provides a turn of the main-chain, which enables the remaining Ca^2+^ ligands to take up coordinating positions, and a hydrogen bond between Gly1145 and Phe1143, which helps to stabilize the otherwise unfavorably close proximity of the negatively charged oxygen atoms in the co-ordination sphere of the bound Ca^2+^ (Figure 4B). Hypothetical three-dimensional (3-D) structural analysis revealed that the distance from Ca^2+^ to Glu1161 (2.85 Å), Asn1176 (2.79 Å), Ile1159 (5.73 Å), and Asp1158 (3.19 Å) changed into 2.54 Å, 2.81 Å, 5.74 Å, and 3.20 Å, respectively, when the mutation replaced Gly1145 with Asp1145 (Figure 4). The G1145D mutation could damage the conformation of residue side chain and produce steric clashes with spatially adjacent residues, causing structural destabilization. A three-dimensional structural analysis also revealed that Gly1145 formed an H bond with Phe1143 and that Glu1161 formed an H bond with Ser1180. When the mutation replaced Gly1145 with Asp1145, these two H bonds were destroyed (Figure 4). The p.G1145D mutation was able to influence the chelating residues of the loop in two ways, the first based on a linear position and the second on the tertiary geometry, and further change the integrity of the structure and function for the *FBN2* protein.

## 3. Discussion

In the present study, we sequenced the genomic DNA (gDNA) of two patients of the CCA pedigree using next-generation sequencing (NGS) and identified the novel heterozygous mutation c.3434G>A (p.G1145D) in the *FBN2* gene, which leads to the folding destabilization of the cbEGF domain for fibrillin-2. We searched the ESP6500, HGMD, dbSNP, TGP, ExAC, and ClinVar database and whole genome sequencing data of 81 unrelated people, and we did not find this mutation. We also did not find this mutation in our 200 unrelated control samples. These results excluded the possibility of a SNP and confirmed that it was a novel mutation. This variant was predicted to be damaging based on SIFT, PolyPhen-2, MutationTaster, REVEL, and M-CAP analysis. The mutation was found to co-segregate in the whole family by Sanger sequencing. Therefore, we confirmed that the p.G1145D mutation in the *FBN2* gene was the causative mutation for this CCA pedigree.

The fibrillin-2 is comprised of 2912 residues and contains 3 EGF domains, 9 TB domains, and 43 cbEGFcb domains. Each cbEGF-like domain of *FBN2* is maintained by six conserved cysteine residues, which form three disulfide bridges to support protein stability (Figure 3B). Ca^2+^ binding in a negatively charged cavity improves fold stability and helps to secure the relative orientation of two neighboring cbEGF domains, composed of a typical sheets–loop–sheets structural unit: two antiparallel β-sheets bridged by a Ca^2+^ chelation loop (Figure 4A).

Mutations in *FBN2* gene can be classified into three groups depending on the residue affected [13,14]. Mutations affecting cysteine residues are likely to alter disulfide bond formation, thereby disrupting the correct fold. Mutations affecting residues in the calcium binding consensus sequence are likely to reduce calcium binding affinity, leading to structural destabilization, and mutations affecting some residues will impair the folding of cbEGF. In the present study, the G1145D substitution disrupts the turn of the main-chain that enables the remaining Ca^2+^ ligands to take up coordinating positions and the hydrogen bond between Gly1145 and Phe1143 that helps to stabilize the otherwise unfavorably close proximity of the negatively charged oxygen atoms in the co-ordination sphere of the bound Ca^2+^, thereby likely impairing the calcium-binding affinity. This mutation could impair the conformation of the residue side chain and produce steric clashes with spatial adjacent residues, causing folding destabilization. Hypothetical three-dimensional structural analysis also showed that G1145D disrupted the formation of the H bonds (Figure 4). The G1145D could change the structural integrity and stability of fibrillin-2, suggesting that this amino acid plays an important role in the structure and function of fibrillin-2. Nevertheless, our analysis is based on the homology-modeled structure. The structure of fibrillin-2 cbEGF pairs should be further investigated by a solution–nuclear magnetic resonance (NMR) method.

The analysis of the distribution of all the previously reported mutations along the *FBN2* gene does not allow us to identify any clear genotype–phenotype correlation. Different types of mutations may have variant phenotypes in CCA patients. There is aortic phenotype variability even among CCA patients with the same *FBN2* mutation. The reasons for the phenotypic heterogeneity caused by *FBN2* mutations are still unknown, and more studies should be further conducted to investigate the mechanism of *FBN2* mutations for CCA. Fibrillins are cysteine-rich glycoproteins that polymerize extracellularly as parallel bundles of head-to-tail monomers and form microfibrils, in association with other proteins, such as latent transforming growth factor beta binding proteins (LTBPs), elastin microfibril interface-located proteins (EMILINs), microfibril-associated glycoproteins (MAGPs), microfibril-associated proteins (MFAPs), and fibulins. The complex combinatorial interactions among these proteins suggest that additional mutational analysis of other genes may give us insights into which specific proteins could modify the functional consequences of the *FBN2* missense mutations that enhance the degree of pleiotropy and variability observed in CCA. However, we analyzed the original WES data and found that the affected patients did not carry any other tissue disorder-associated mutations. Unfortunately, most studies of CCA usually use traditional Sanger sequencing to analyze only the *FBN2* mutations and lack mutational information for other genes. With the advent of NGS technology, including exome sequencing and whole-genome sequencing, more novel gene mutations or variants resulting in CCA will likely be discovered. The unveiling of the genes and pathways involved in the development of CCA will help to explain its diverse clinical phenotypes and strong genetic heterogeneity, and may contribute to developing targeted therapies for this condition.

In summary, our study identified a novel *FBN2* mutation (p.G1145D) as the disease-causing mutation of CCA. Exome sequencing provides a powerful and highly efficient approach for identifying disease-causing gene of CCA, while excluding genes responsible for phenotypically similar disorders, such as MFS. Our findings may also provide new insights into the cause and diagnosis of CCA and may have implications in genetic counseling and clinical management for families with CCA.

## 4. Materials and Methods

### 4.1. Ethics Statement

The Ethics Committee of the Shanghai Children’s Medical Center reviewed and approved this study (SCMC-201015). Written, informed consent was obtained from the patient’s parents.

### 4.2. Genomic DNA Preparation

gDNA was extracted from peripheral blood samples using a QIAamp DNA Blood Mini Kit (Qiagen, Hilden, Germany). A spectrophotometer NanoDrop 2000 (NanoDrop Products, Wilmington, Delaware, USA) was used to determine the purity and concentration of the gDNA in the sample. The gDNA library was prepared using the TruSeq DNA Sample Preparation Kit (Illumina, San Diego, CA, USA) in accordance with the manufacturer’s instructions.

### 4.3. Whole Exome Capture and Sequencing

We sequenced the gDNA of two patients (the proband and her mother) of the syndactyly pedigree using WES. In-solution exome enrichment was performed using the TruSeq Exome Enrichment Kit (Illumina, San Diego, CA, USA) according to the manufacturer’s instructions. The enriched DNA samples were sequenced via 2 × 100 paired-end sequencing using a Hiseq2000 Sequencing System (Illumina). Illumina Sequencing Control v2.8, Illumina Off-Line Basecaller v1.8, and Illumina Consensus Assessment of Sequence and Variation v1.8 software were used to produce 100 base pair (bp) sequence reads.

### 4.4. Analysis of Sequencing Data

Sequence reads were aligned to the human reference genome (hg19) using a Burrows–Wheeler Aligner and Samtools with default parameters. Variants were identified using the Genome Analysis Toolkit (GATK) [15] and VarScan software [16]. Coverage analysis was determined using the Picard software Calculate HsMetrics tool. Reads that matched exonic regions, including exon–intron boundaries, were analyzed. Single nucleotide variants (SNVs) and insertions/deletions (Indels) were analyzed using different filtering steps. Genes with at least one heterozygous changes in the DNA sequence were considered to be the most likely to cause disease. Annovar [17], a software tool that accesses and utilizes information from external databases to assess the implications and consequences of a given sequence alteration, was used to annotate the resulting list of variants.

The variant detection frequency was set at a minimum of 20% of the reads covering any aberration. A minimum coverage of 10 reads was set as the threshold for any variant to be considered a real mutation. In each case, all variants listed in the most recent version of the National Center for Biotechnology Information (NCBI) single nucleotide polymorphism (SNP) database (dbSNP) were excluded. Silent mutations were also excluded. Low-frequency frameshift and truncating mutations were considered pathogenic. Unreported non-synonymous amino acid variants were analyzed using Polyphen-2 [18], SIFT [19], MutationTaster [20], REVEL [21], and M-CAP [22] to assess any potentially damaging effects. The exome variants were filtered based on co-segregation with a model of an autosomal dominant disorder. In addition, genes annotated to be associated with skeletal disorders were prioritized manually. Variants that passed these filtering steps were considered to be most likely to cause disease and were subsequently validated by Sanger sequencing.

### 4.5. Mutation Validation by Sanger Sequencing

All available family members were tested for co-segregation of the candidate CCA-causing variant with the disease phenotype by Sanger sequencing. PCR primers were designed to contained the mutation sites and their flanking regions (PCR primers, PCR reaction conditions are available upon request) using Primer3 [23]. An ABI Prism 3730xl DNA Sequencer and the Big Dye Terminator Cycle Sequencing Ready Reaction Kit 3.1 (Applied Biosystems, Foster City, CA, USA) were used for sequencing. Sequences were assembled and analyzed with Mutation Surveyor software (SoftGenetics, State College, PA, USA). We also tested the DNA of 200 healthy controls.

### 4.6. Molecular Modeling of the FBN2 Protein and Conservation Analysis

Domain structure of fibrillin-2 was recovered using Simple Modular Research Tool (SMART) [24]. The search for structural repeats and the multiple sequence alignment of Calcium-Binding Epidermal Growth Factor (cbEGF) Motifs 12–13 were performed using Rapid Automatic Detection and Alignment of Repeats (RADAR) [25]. To evaluate the evolutionary conservation of the mutated site, protein sequences of fibrillin-2 from ten animal species from fishes to mammals, including human (Homo sapiens: NP_001990.2), mouse (Mus musculus: NP_034311.2), rat (*Rattus norvegicus*: NP_114014.1), dog (*Canis lupus*: XP_013973170.1), cattle (*Bos taurus*: NP_001265517.1), pig (*Sus scrofa*: XP_013850528.1), chicken (*Gallus gallus*: XP_004949436.1), gekko (*Gekko japonicus*: XP_015261270.1), frog (*Xenopus laevis*: XP_002931771.2), and zebrafish (*Danio rerio*: NP_001129262.1) were aligned using Clustal W embedded in MEGA 7 [26].

The initial mutant variant structures for the cbEGF Motifs 12–13 (Residues 1115–1199) were constructed using the automated protein-homology modeling server SWISS-MODEL [27], using protein structure of a cbEGF-like domain pair from the neonatal region of human fibrillin-1 as a structural template (PDB: 1LMJ). PROCHECK was employed to estimate the quality of our models. There are 84.0% residues located in the “core” and “allowed” regions, 15.9% in the “general” region and only 0% in the “disallowed” regions. In the computational structure, 100.0% of the bond lengths for the main-chain residues and 100.0% of the bond angles for the main-chain residues are within the allowed limits. The sequence identity between human sequences of fibrillin-1 and fibrillin-2 was 77.9%. Analysis of the 3-D structure of the proteins was carried out using Swiss-PdbViewer [28]. 

## Figures and Tables

**Table 1:** Clinical and genetic data of three patients with congenital contractural arachnodactyly (CCA).

Subject	III-1	III-2	II-2
Gender	Female	Female	Female
Age (years)	15	12	43
Mutation	Gly1145Asp	Gly1145Asp	Gly1145Asp
Genotype	Heterozygote	Heterozygote	Heterozygote
Tall stature	−	−	−
External ear anomalies	−	−	−
Arachnodactyly	+	+	+
Camptodactyly	+	+	+
Contractures of knuckles	+	+	+
Contractures of large joints	−	−	−
Scoliosis	−	−	−
Pectus deformity	−	−	−
Ocular complication	−	−	−
Cardiovascular complication	−	−	−

+, present; −, absent.
